# Outcome Reporting Bias in Government-Sponsored Policy Evaluations: A Qualitative Content Analysis of 13 Studies

**DOI:** 10.1371/journal.pone.0163702

**Published:** 2016-09-30

**Authors:** Arnaud Vaganay

**Affiliations:** London School of Economics and Political Science, London, United Kingdom; Tilburg University, NETHERLANDS

## Abstract

The reporting of evaluation outcomes can be a point of contention between evaluators and policy-makers when a given reform fails to fulfil its promises. Whereas evaluators are required to report outcomes in full, policy-makers have a vested interest in framing these outcomes in a positive light–especially when they previously expressed a commitment to the reform. The current evidence base is limited to a survey of policy evaluators, a study on reporting bias in education research and several studies investigating the influence of industry sponsorship on the reporting of clinical trials. The objective of this study was twofold. Firstly, it aimed to assess the risk of outcome reporting bias (ORB or ‘spin’) in pilot evaluation reports, using seven indicators developed by clinicians. Secondly, it sought to examine how the government’s commitment to a given reform may affect the level of ORB found in the corresponding evaluation report. To answer these questions, 13 evaluation reports were content-analysed, all of which found a non-significant effect of the intervention on its stated primary outcome. These reports were systematically selected from a dataset of 233 pilot and experimental evaluations spanning three policy areas and 13 years of government-commissioned research in the UK. The results show that the risk of ORB is real. Indeed, all studies reviewed here resorted to at least one of the presentational strategies associated with a risk of spin. This study also found a small, negative association between the seniority of the reform’s champion and the risk of ORB in the evaluation of that reform. The publication of protocols and the use of reporting guidelines are recommended.

## 1. Introduction

In its most extreme form, reporting bias refers to the non-publication of a study because of undesirable results (publication bias). Empirical research has consistently shown that published research is more likely to be positive or statistically significant than unpublished research [1]. However, analysing unpublished studies is particularly difficult, as it requires collecting data from sponsors, regulators and the investigators themselves. It would be even more difficult to analyse unpublished policy research, where protocols and registration are not required. Thus, this paper had to pursue a different strategy.

Within-study outcome reporting bias (ORB or ‘spin’) relates to studies that have been published. It has been defined as a specific reporting strategy, emphasizing the beneficial effect of an experimental treatment or intervention [2]. The use of spin in scientific writing can result from ignorance of the scientific issue, unconscious bias, or wilful intent to distract the reader from statistically non-significant results [2,3]. Spin can take different forms, such as selective reporting, a particular focus on less informative results or an inadequate interpretation of non-statistically significant differences [2]. Spin can also occur at later stages, for example in the communication of results to stakeholders and the media [4,5]; however this particular aspect of spin is not addressed here.

Spin leads to overestimate the effect of the intervention. This matters for many reasons. Firstly, the interventions may have insignificant or even harmful effects on their subjects. Secondly, voters using this type of information to appraise government performance will be misled. Thirdly, researchers and policy-makers using these results to inform subsequent policies will also be misguided [3,6].

The issue of reporting bias is well documented in the literature: the term was found in the title of 148 documents referenced by the Web of Science and 37 documents referenced by PubMed Central as of February 2016. Excluding cases not related to the reporting of research outcomes (e.g. news reporting, financial reporting, etc.), the overwhelming majority of this literature is related to the biomedical sciences. This literature includes studies analysing the prevalence of ORB in drug trials [7–11], and disciplines such as acupuncture [12], cognitive sciences [13], and intensive care medicine [14]. It also includes cross-disciplinary reviews [1,2,15–19], studies on the possible causes of ORB [20], its effects on the interpretation of results by health practitioners [21] as well as interviews and surveys of authors [22,23]. Methods have been devised to detect ORB [24–26] and estimate its effect on confidence intervals and p-values in meta-analysis [27,28]. Possible solutions have been discussed [29–31] and evaluated [32,33]. Books have alerted the wider public on the practice of ORB [34,35].

This study makes three main contributions. Theoretically, it offers a comprehensive theory of ORB, which encompasses its possible causes, effects and moderating factors. This approach departs from previous research focusing mainly on the manifestations of ORB. Identifying possible causes allows the formulation of solutions. Empirically, this study builds on the only study of ORB in the area of social sciences [36]. It addresses two questions: (1) What is the prevalence of ORB in government-sponsored social policy evaluation? (2) Were evaluations of reforms to which the government was committed more likely to be spun than others? Methodologically, this study is the first of its kind using qualitative content analysis. This method, based on text, gives readers concrete examples of the type of language associated with spin. It also tests the feasibility of a quantitative study in the area of government-sponsored evaluation.

## 2. Theoretical Framework

The theory underpinning this paper is that ORB is a human behaviour, which can be corrected or ‘nudged’. The following section justifies this theory.

### Effect of ORB

ORB can take many forms. This paper focuses on seven of them. A first strategy consists of ‘filtering out’ the least convenient results [37–39]. This is only noticeable through systematic comparisons between a final study and its protocol [38,39], earlier versions of the same study [23,36] or educated guesses based on the available data [37]. There is strong evidence of an association between the size and significance of an effect and the odds of being fully reported. Overall, favourable and significant effects are more likely to be (fully) reported [1].

The second strategy consists of reporting outcomes superficially [1]. Direct evidence of such bias has recently been shown in two cohort studies that compared trial publications with the original protocols [38,39].

The third strategy consists of overemphasising favourable results or underemphasising unfavourable results. This risk of ‘interpretative bias’ occurs mainly when a study shows a difference that is not statistically significant [40,41].

The fourth strategy consists of conducting separate analyses for intervention and control groups. The essence of a clinical trial or policy pilot is to compare the outcomes of groups of individuals going through different interventions. We expect studies to give us an estimate of the difference with a confidence interval and a P-value. However, rather than comparing the groups directly, researchers sometimes look *within* groups at the change between the outcome measure from pre-intervention baseline to the final measurement at the end of the trial. They then perform a test of the null hypothesis that the mean difference is zero, separately in each group. They may then report that in one group this difference is significant but not in the other and conclude that this is evidence that the groups, and hence the treatments, are different [42].

The fifth strategy consists of spurious subgroup analyses. The effects of an intervention on the entire study population are of primary interest in a study. It could be appealing, however, for investigators and research commissioners to identify differential effects in subgroups based on characteristics of trial participants or interventions. This analytic approach, termed ‘subgroup analysis’, can sometimes be informative–but it is often associated with a risk of false-positive results [25,43–45]. Some have compared them as data-driven ‘fishing expeditions’ [46,47]. Even when investigators specify a limited number of subgroup analyses *ex ante*, chance can result in the identification of spurious subgroup effects [25,44,46,47].

The sixth strategy consists of upgrading or downgrading outcomes. The primary outcome of a study is the outcome of greatest importance. Data on secondary outcomes are used to evaluate additional effects of the intervention. When a change in outcomes occurs, it must be said and justified [48,49].

The seventh and last strategy is conclusion bias. One can look at the evaluator’s final judgement of the merit of the intervention in the conclusion of the report or its executive summary. An overemphasis on positive results will be taken as an indication of ORB.

### Causes of ORB

ORB illustrates a cognitive dissonance between the will to conform to the norms of science and the will to honour other commitments. Thus, ORB is more likely to occur in scientific disciplines where the norms of reporting are relatively weak. There is strong evidence that this is the case in social sciences in general and in social policy research in particular. This is visible at the peer review stage, where the quantity and quality of information of a given study is externally assessed. Incomplete reporting has been found a major factor of rejection of manuscripts [50–52]. Many authors have expressed their frustration with regards to the inconsistency of the peer review process [53]. Further down the editorial line, journal referees do not seem to comply with any kind of reporting norm. In general, their strictness has been found to be associated with individual characteristics such as gender [54], disciplinary affiliation [55], cultural background [56] and personal preferences [57]. Some reporting guidelines are increasingly endorsed. Those include the *Consolidated Standards for Reporting Trials* (CONSORT) [58], the *Preferred Reporting Items for Systematic reviews and Meta-Analyses* (PRISMA) [59] and the *Strengthening the reporting of observational studies in epidemiology* (STROBE) [60]. However, these guidelines are aimed at clinicians, and are rarely used by social scientists.

ORB is also more likely to occur when the incentives to deviate from the norms of scientific reporting are relatively strong. There are (at least) two such incentives. The first incentive is a public commitment to a pre-determined outcome. This commitment can be ideological (i.e. a preference for one policy over another) or path-dependent (i.e. dictated by previous decisions or previous research findings). Such commitments can have very strong effects on behaviours [61,62]. The second incentive is the urge to reciprocate. This situation occurs when the researcher acts as the agent of a principal who (i) is less committed to the scientific norms of science than the researcher is; and (ii) can reward the researcher for deviating from the norms of reporting. This is relevant to this paper. Indeed, an increasing share of research is being conducted outside of academia or commissioned by non-academic organisations, such as businesses, governments and interest groups [63,64].

The underlying assumption in medical meta-research is that the effect of ‘non-scientific’ incentives on reporting is moderated by the salience of the disease or the financial returns expected from the new treatment. The investments made for the development of new drugs are such that pharmaceutical companies sometimes cannot afford reporting on useless or harmful drugs. This paper investigates whether a similar risk of bias exists in policy research.

## 3. The Case at Hand

This study analyses the content of a small sample of evaluations commissioned by the British government between May 1997 and May 2010. This period corresponds to the Labour governments of Tony Blair and Gordon Brown. To assess the credibility and generalizability of the conclusions below, it is imperative to briefly describe the institutional and cultural context in which these evaluations were conducted.

In the UK, the vast majority of policy evaluations are carried out by contracted organisations on behalf of ministerial departments such as the Department for Work and Pensions (DWP), the Home Office or the Department for Education (DfE). Therefore, evaluators and policy-makers must come to an agreement on *what* information should be reported and *how* this information should be reported [63–65]. This situation creates a cognitive dissonance. On the one hand, evaluators are recruited based on their reputation for competence and expertise. On the other hand, they might want to reciprocate the favour of having been awarded a contract and maximise the chances of winning the next [64].

When it comes to reporting the findings of an evaluation, the tension might be less strong than expected. On the one hand, prescriptions for the reporting of research outcomes are minimal. The reference document for the management of research projects, the *Magenta Book* [66], only provides a few ‘tips’ to report research findings. This is in stark contrast with biomedical research, where reporting norms are much stricter. On the other hand, the pressure to produce results that are favourable to the sponsor can be strong. A recent survey of academics having completed commissioned research for the UK government found that more than half of respondents reported that they were asked to make significant changes to their draft reports (i.e. changes affecting the interpretation of findings or the weight given to them). Several interviewees indicated that policy-makers were more inclined to try and influence the reporting of outcomes when the reform was perceived as politically salient [64]. This is consistent with the idea that policy-makers are rarely neutral about their research. If they are testing a novel intervention, they usually suspect that it is effective otherwise they could not convince themselves and their stakeholders that it is worth evaluating. This lack of equipoise can affect the way they interpret negative results. Having invested a large amount of political capital in developing a reform, policy-makers might find it difficult to accept that it is ineffective or even harmful.

## 4. Materials and Methods

### Approach

The design of this study was shaped by the various constraints pertaining to the research question and the available data. To begin with, the definition of reporting bias presented in the introduction implies that studies not supporting the initial working hypothesis (i.e. the intervention has a positive and significant effect on the population) are more likely to be spun than others [40]. Thus, the studies reviewed in this paper all reported a primary outcome that was either not statistically significant at the conventional level (P≥0.05) or in the direction opposed to the initial hypothesis (i.e. the intervention has a negative effect).

The availability of data created additional constraints. Firstly, the fact that policy evaluations are overwhelmingly commissioned by the governments which designed and implemented those policies means that it is not possible to compare the amount of reporting bias in studies sponsored by the government with studies sponsored by non-governmental bodies. Such design would have provided a useful counterfactual. Although it has been used in medical research to assess the effect of industry sponsorship on reporting [7], it remains difficult to replicate in policy research. Instead, this study contrasted interventions with different levels of policy commitment.

Secondly, the absence of formal research protocols for the evaluation of public policy means that it is not possible to estimate the amount of reporting bias through systematic comparisons between the content of published reports and that of protocols [7] or research proposals [10], though a similar strategy was attempted (see below). In other words, there is no clear baseline against which published results could be systematically compared. Instead, this study looked for evidence of research decisions that have previously been associated with an intention to spin. Those include incomplete statistical outputs, spurious analyses and biased interpretations of results. Those variables are presented in greater details below.

Thirdly, the number of evaluation reports amenable to this kind of research was too limited to allow a quantitative analysis. Instead, a qualitative approach was adopted, focusing on the content of these reports, their claims and the language adopted by evaluators. Three sections were analysed: the ‘results’ sections, the study conclusions and the ‘executive summaries’ (although the latter two sections proved nearly identical). The main implication for this study is that my observations are limited to the chosen sample.

In sum, this paper offers a qualitative analysis of the content of 13 evaluation reports with different levels of policy commitment. Its objective is to find out, in the context of studies with non-significant primary outcome, whether a stronger commitment to the policy is qualitatively associated with more spin.

### Selection process

**Fig 1:**
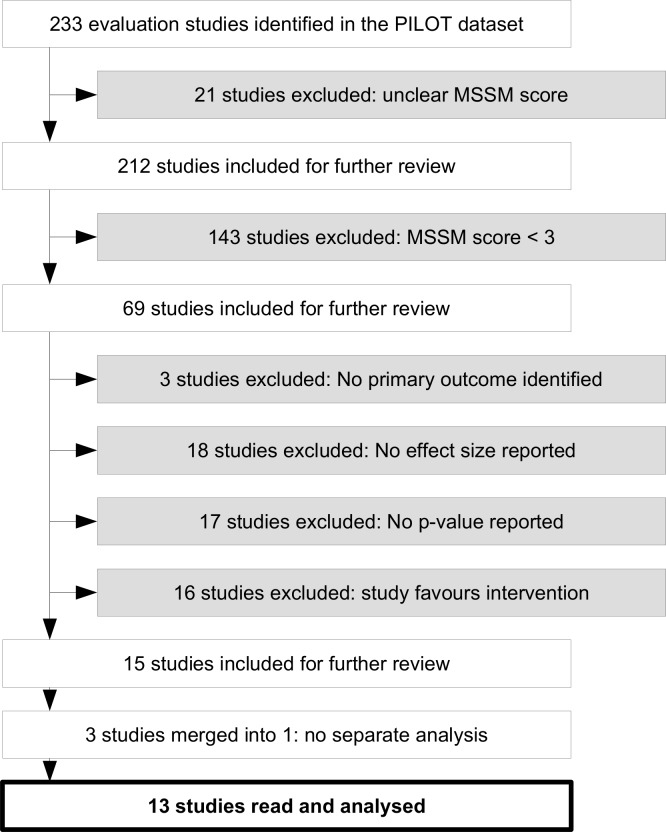
Flow chart of the process of identifying and selecting evaluation reports.

The 13 studies analysed in this paper were selected from the PILOT dataset, which is publicly available (https://osf.io/9bqkv/), along with its codebook (https://osf.io/ph76n/). The selection process followed a number of steps (Fig 1). First, studies with a score of 3 and above on the Maryland Scale of Scientific Method were included and studies with a ‘weaker’ design were excluded (i.e. studies not including a counterfactual) [67]. When several studies were available for the same pilot, the one offering the most definitive conclusions regarding the effect of the intervention (*e*.*g*. final report as opposed to interim report) was selected. From this sample, the full-text studies were screened and their primary outcomes identified. Only studies showing that the intervention had a non-significant effect were selected (P≥0.05). The decision to use a P-value of 0.05 or a 95% confidence interval to determine statistical significance is arbitrary but widely accepted. Conversely, studies for which the primary outcome could not be identified with confidence and studies showing a positive and significant effect of the intervention were excluded. In one study, the primary outcome was not identified from the evaluation report itself but from the technical specifications (TS) issued by the sponsoring department for the evaluation of the intervention. The selection process resulted in a sample of 15 studies. However, three of these studies (CONNECT, REMEDI and Justice Research Consortium) were analysed simultaneously in a single report, so it was decided to treat them as a single study. Thus the final sample includes 13 studies.

In addition, Freedom of Information requests were sent to the relevant government departments to get hold of the TS issued for these evaluations, as well as any interim report not published on their respective websites. TS were obtained for two studies out of six (DWP-1; DFE-1) and one interim report for one study only (three studies had no interim report). This interim report was screened but no impact analysis was found and so it was decided not to include it in the study corpus. The list of studies that were reviewed and TS can be found in Table 1. A summary of each intervention is available at: https://osf.io/5vu4s/. In the remainder of this article, each study is referred to by its department and a number (e.g. DWP-1).

**Table 1:** References of studies included in the corpus of studies.

**Code**	**Final reports**	**Ref. number**	**Document URL**
DWP-1	Pathways to Work	[80]	https://osf.io/uy85g/
DWP-2	Job Retention and Rehabilitation Pilot	[81]	https://osf.io/f53hd/
DWP-3	Jobseeker’s Allowance Skills Conditionality Pilot	[82]	https://osf.io/98psd/
DWP-4	Jobseekers Allowance Intervention Pilots	[83]	https://osf.io/fkg2e/
DWP-5	ONE Pathfinder	[84]	https://osf.io/zh6ws/
DWP-6	StepUP Pilot	[85]	https://osf.io/w8pk9/
DFE-1	Early Education Pilot for Two-Year-Old Children	[86]	https://osf.io/48byz/
DFE-2	The Care Placements Evaluation (CaPE)	[87]	https://osf.io/f5mex/
DFE-3	Every Child a Writer	[88]	https://osf.io/z4egn/
DFE-4	Empowering Young People Pilots (EYPP)	[89]	https://osf.io/6bwy4/
HOME-1	Alcohol Arrest Referral Pilot Schemes (Phase 2)	[90]	https://osf.io/tud5v/
HOME-2	Three restorative justice pilots: JRC, REMEDI and CONNECT	[91]	https://osf.io/npfvz/
HOME-3	Judicial mediation in Employment Tribunals	[92]	https://osf.io/pa4b7/
**Code**	**Technical specifications**	**Ref. number**	
DWP-1-TS	Pathways to Work	Unpublished	https://osf.io/wf9c3/
DFE-1-TS	Early Education Pilot for Two-Year-Old Children	Unpublished	https://osf.io/syr9n/

### Indicators of ORB

The seven indicators of spin mentioned in section 2 were operationalized as follows. *Missing outcomes* were recorded based on a comparison between the TS for a given study (used as protocol) and the corresponding final report [38,39]. As a given outcome can be operationalized in many different ways, an ordinal scale was created, measuring the strength of the correspondence (or agreement) between the outcomes planned in the TS and those reported in the final report. Thus, for each outcome listed in the TS, the correspondence was considered strong if the outcome was also reported in the final report. Conversely, the correspondence was considered non-existent when an outcome was planned in the TS but could not be found in the final report. The correspondence was considered weak when an outcome was present in both documents but operationalized in a different way (e.g. the variable was measured at a different time or using a different definition).

*Incomplete reporting* was recorded when, for each identified outcome, one or more of the following three elements was missing: (a) group numbers; (b) size of intervention effect; and (c) a measure of precision/variability (P-value and/or confidence interval).

*Interpretative bias* was recorded when investigators claimed that the non-significant result was due to lack of power rather than lack of effect. It is apparent when the investigators claim that an effect is “borderline significant” or state that no firm conclusions can be drawn because of the modest sample size. In contrast, investigators may downplay the fact that a study shows a non-significant effect that opposes the study hypothesis by emphasising the results are not statistically significant [40]. For the purpose of this analysis, a non-significant result was defined as a regression coefficient with P-value larger than the conventional 5% level.

*Spurious within-group effects* were recorded in cases where investigators had (i) performed before-after analyses separately for the intervention group or pilot region and (ii) drawn conclusions about the effect of the intervention [42].

The prevalence of *spurious subgroup effects* was determined using five criteria developed by clinicians [44]. These include for example whether the hypothesis of a subgroup effect preceded the analysis, and was based on an interaction rather than on separate analyses. These criteria are listed in section 5.

The prevalence of *upgraded/downgraded outcomes* is usually established by comparing the order of the outcomes in the research protocol and in the published study. As this was not possible in this project, the comparison was made within studies, i.e. by comparing the order of results in the executive summary and the ‘results’ section. Four situations were considered: (1) a primary outcome is downgraded to secondary (downgrade); (2) a secondary outcome is upgraded to primary (upgrade); (3) a new outcome not stated in the protocol is added to the full review (addition); or (4) an outcome stated in the protocol was omitted from the full review (omission) [48,49].

The prevalence of *conclusion bias* was determined using an existing classification [2]. However, this assessment was based on executive summaries rather than conclusions, as those tend to be more widely read, especially among non-specialists. *High spin* was defined as the suggestion that, overall, the intervention was a success despite a non-significant primary outcome. *Moderate spin* was defined as the acknowledgment of the non-significant effect of the intervention, but with an immediate emphasis on spurious analyses meant to distract the reader from the main study outcome. *Low spin* was defined as the acknowledgment of the statistically non-significant result for the primary outcome.

### Endorsement of the reform

Extra information was collected for each study to measure the political salience of the reform. In this paper, the level of seniority of the ‘champion’ or ‘sponsor’ of the reform was used as proxy. This was determined based on who first announced the pilot. The announcement of a pilot can be seen as a delegation issue, whereby each principal, from the Prime minister to a civil servant can decide whether to be the ‘manager’, taking direct responsibility for the outcome, or the ‘chair of the board’ overlooking operations [68]. Given politicians’ propensity to avoid blame even when that implies not getting credit [69], it was considered that a pilot announced by the Prime minister was more politically salient than a pilot announced by any other member of the Cabinet (Chancellor, Senior Minister, Junior Minister) or a pilot not announced at all. An ordinal variable reflecting these categories (From 1: Not announced to 5: Announced by the Prime Minister) was created for this analysis using information provided in the reports and by *WiredGov*, the main news alert channel for UK government and public sector officials [70].

### Analysis

The research question was answered using summative content analysis (SCA). SCA is one the three approaches to qualitative content analysis [71]. SCA starts with identifying and quantifying certain words or content, using an existing theoretical framework, such as the typology of ORB. This quantification is not an attempt to make inferences but, rather, to explore usage. SCA also requires a qualitative interpretation of this content, for example to assess the extent to which the analyses reported in a final report match the initial objectives of the researcher/policy-maker [71]. In this paper, SCA was performed in three steps. First, the number of occurrences of a type of ORB was counted using pre-existing definitions. On some occasions, this was straightforward (e.g. assessing whether sample sizes were reported in statistical outputs), on others it required an informed judgement (e.g. assessing whether a given outcome was the same in a protocol and in a final report). In the latter case, the decision criteria were defined as clearly as possible. Second, an overall risk of spin was calculated for each study. Third, this risk was correlated with the seniority of the policy-maker endorsing the reform.

The analysis was done three times a year apart, to assess intra-coder reliability. The first analysis was carried out in the summer of 2013, the second one in the autumn of 2014 and the third one in October 2015. The second analysis led to more accurate results: whereas all instances of spin identified in the first analysis were also identified in the second analysis, the second analysis found an instance of spin that had been previously missed. Seven additional studies were added between the second analysis and the third analysis. No discrepancy was found in the coding of the six ‘original’ studies between round 2 and round 3. However the operationalization of a ‘missing outcome’ was changed from a binary code (the outcome is present/absent in the final report) to an ordinal one (the correspondence between a given outcome in the protocol and the final report is strong/weak/non-existent). A new correspondence diagram was also produced.

In addition to recoding, steps were taken to make this study reproducible. All materials (datasets, units of analysis, tables, figures, textual evidence) can be found on the Open Science Framework (https://osf.io/qyjwc/).

## 5. Results

### Prevalence of ORB

**Fig 2:**
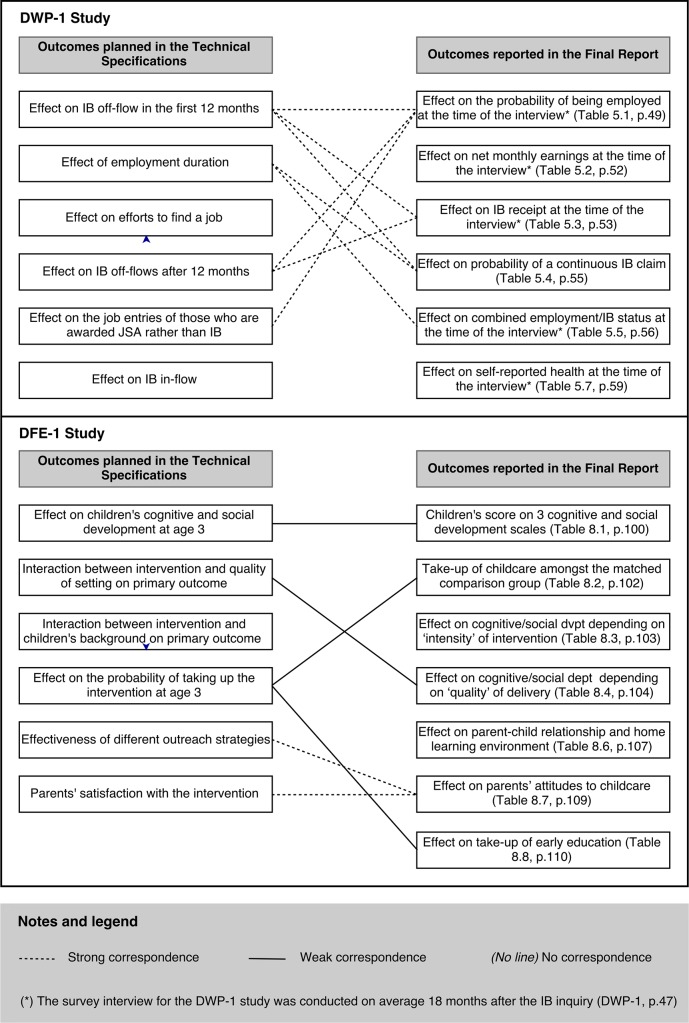
Correspondence diagram: Outcomes as planned in technical specifications vs. outcomes as reported in final reports (for studies DWP-1 and DFE-1).

The prevalence of ORB through the omission of outcome indicators could not be assessed across the sample. Only two sets of TS could be obtained for two studies, DWP-1 and DFE-1. This section summarises the results of the analysis presented in section 4 above for these two studies only. A correspondence diagram (Fig 2) summarises the evidence. Looking at study DWP-1 (upper panel), one can see a list of six outcomes in both the TS and the final report. However, the correspondence between these two sets of outcomes is less than perfect. Indeed, of the six outcomes listed in the TS: none was reported as such in the final report; two could not be found at all (effect of the intervention on efforts to find a job; effect on Incapacity Benefit [IB] inflow); and four were considered weakly related to an outcome in the final report. One of those is the effect of the intervention on IB off-flows in the first 12 months of the programme. This outcome was considered weakly related to three outcomes found in the final report. For example, it is related to the effect of the intervention on the probability of being employed 18 months after the start of the intervention only to the extent that (i) all clients terminate their IB claim because they found a job; and (ii) the effect of the intervention is the same between the 12^th^ and the 18^th^ month. Looking at study DFE-1 (lower panel), one can see a list of six outcomes in the TS and seven outcomes in the final report. Out of the six outcomes in the TS: three were reported as such in the final report; one could not be found at all; and two were considered weakly related to an outcome in the final report. Interestingly, in both study DWP-1 and DFE-1, two outcomes were reported in the final report but were not planned in the TS. Thus, the correspondence diagram shows that there is a risk of ORB through the omission, addition or the modification of outcome indicators, and that the level of this risk might be associated with characteristics such as the policy area or the department commissioning the study.

**Fig 3:**
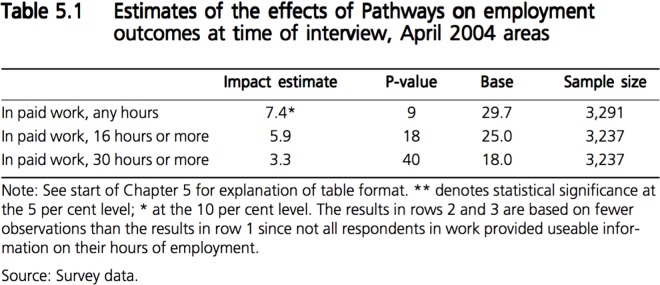
Example of complete reporting (DWP-1, Table 5.1, p.49).

**Fig 4:**
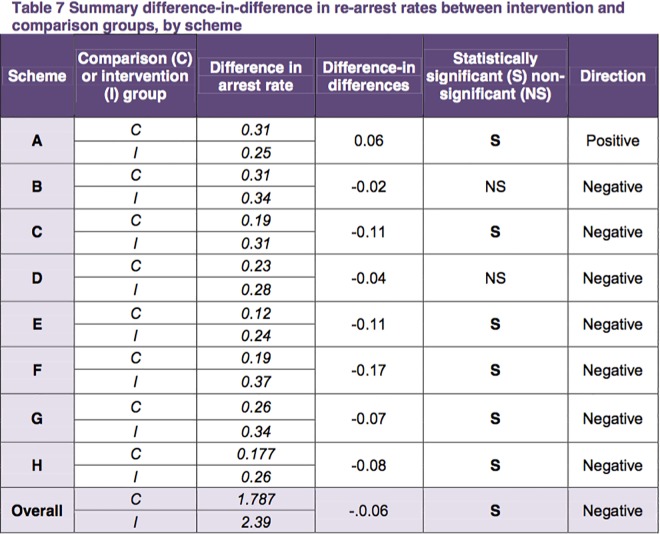
Example of incomplete reporting (HOME-1, Table 7, p.17).

Evidence of incomplete reporting was found in five of the 13 studies (DFE-3; DWP-4; DWP-5; HOME-1; HOME-3). The sample size was the least reported information, with four reports omitting this information or inconsistently reporting it (DFE-3; DWP-4; DWP-5; HOME-3). In two reports, (DWP-4; HOME-3), statistically significant effects were flagged but the significance level used by the investigators could not be found. In one report (DWP-4), the analysis was presented as a series of graphs rather than tables and effect sizes could not be found. Examples of complete and incomplete reporting can be found in Fig 3 and Fig 4 respectively. The lack of clarity and consistence in the presentation of these results, both across studies and within studies, was found to be a major flaw.

Evidence of interpretative bias was found in five of the 13 studies (DFE-4; DWP-1; DWP-4; HOME-2; HOME-3). Among those studies, three types of interpretative bias were found. In two studies (DWP-4; HOME-3), conclusions regarding the statistical significance of the intervention’s effects were drawn without any evidence. In one study (DWP-1), the statistical significance of the effect of the intervention on the primary outcome was based on a ‘looser’ scale than in the rest of the study (P = 0.05). Quote 3 in Table 2 shows that a P-value of 0.09 was considered statistically significant. The footnote justifying this decision (Quote 4) speaks volumes. In this case, using the “smaller sample size” to justify the decision to regard a P-value of 0.09 as “significant” could be seen by many as disingenuous, given that this sample size was 3,291 (DWP-1, Table 5.1, p.49). As a comparison, the analyses performed in the DWP-2 study were all based on samples of less than 3,000 individuals and yet used P-values of 0.05 as cut-off point. This interpretation of the effect of *Pathways to Work* is a prime example of spin: factually correct but unconventional (including with regards to the department’s own rules) and conveniently supportive of policy-makers’ initial hypothesis. In two studies (DWP-1, HOME-2), the insignificant effect of the intervention was attributed to a lack of statistical power rather than to the ineffectiveness of the intervention (Quote 5). Quote 6 provides an example of clear and straightforward interpretation.

**Table 2:** Quotes from the corpus of studies.

Number	Quote	Reference
1	“A key requirement underpinning sampling is the need to include a discussion on the capability of analysing subgroups, and any implications for overall samples of the need to estimate impacts of separate components. We would welcome suggestions on types of subgroup analyses”	DWP-1-TS, p.17
2	“[Tenderers] must also demonstrate a commitment to meet deadlines and yet be sufficiently flexible, should the programme of work require amending”	DWP-1-TS, p.26
3	“The P-value suggests that the impact is statistically significant since there is only a nine per cent probability of finding an effect of this size by chance”	DWP-1, p.48
4	“By convention, P-values of five per cent or less are regarded as indicating statistical significance. However, this is essentially arbitrary and ignores the continuous nature of P-values. The approach taken in this report is to use the conventional five per cent P-values for the results based on the administrative data but to use ten per cent P-values for the results based on the survey data in view of the smaller sample size available for these estimates”	DWP-1, p.48
5	“The small sample size of those in work and with earnings information at the time of the outcome interview reduced the likelihood of detecting an impact on earnings. No statistically significant impact of Pathways on monthly net earnings about a year and a half after the initial incapacity benefits enquiry was found (Table 5.2) (…). In view of the employment effect of Pathways, one would expect a positive impact on earnings”	DWP-1, p.2
6	“The finding is clear-cut: there is no evidence that, on average, the pilot improved the non-verbal reasoning of children overall”	DFE-1, p.99
7	“The choice of variables from which to create sub-groups is somewhat arbitrary. The final list is based on a selection of possible variables for which: (i) the sub-groups have large enough sample sizes for at least moderately large impacts to be detected; (ii) there is some expectation that impacts may have been different in at least some of the sub-groups”.	DWP-2, p.49
8	“The quantitative analysis used administrative data to provide details on the implementation of the pilot and whether it could be used to provide valid estimates of the impact of mandation”	DWP-3, p.1
9	“Overall, the results are encouraging in that they suggest Pathways continues to have a positive impact on employment and, furthermore, that this impact may be sustained”	DWP-1, p.4
10	“This report has shown no evidence that offering Job Retention and Rehabilitation Pilot interventions to those off work sick improved their chances of returning to work”	DWP-2, p.129
11	“Tenderers’ suggestions for evaluating net impact needs to be of the highest quality, and this will be looked at specifically in addition to a more broad requirement of methodological expertise”	DWP-1-TS, p.27
12	“The contractor will be expected to work closely with officials of the Department throughout the research, keeping them informed of progress and involving them in key decisions. Officials in policy and analytical branches in DWP and DH must have the opportunity to comment on and approve topic guides and questionnaires, formats for analysis and draft reports”	DWP-1-TS, p.22-23
13	“This will be a high-profile evaluation and to get full value from it, timely and high quality reporting is essential. To ensure full value of the evaluation tenderers should consider ways in which emerging findings from studies can most appropriately be fed back to policy officials in order to inform further policy development. For example in advance of the production of draft reports, contractors are likely to be asked to present headline findings to core policy officials and analysts”	DWP-1-TS, p.24
14	“It is the expectation that the key outputs from the study will be in the public domain. The Department will aim to publish key outputs within a reasonable period of time following receipt of an agreed final report. The publication of any research articles or other publications based on information collected for this study will be subject to approval from the [DFE]. However, this will not be unreasonably withheld”	DFE-1-TS, p.4

Evidence of spurious within-group effects was found in two of the 13 studies (DFE-4; HOME-3). In one study (DFE-4), although data was collected in separate pilot and control areas, the evaluators ended up using a different control group in their main analysis. This control group included the non-users of the intervention in the pilot areas (see DFE-4, Table 5.8, p.62). This is a peculiar decision, given that the intervention was available on a voluntary basis. It is doubtful that those who did take up the intervention were comparable to those who did not. To be clear, a separate analysis using data collected in control areas was performed and included in the study as an Annex (see DFE-4, Annex D, p.141). However, the results of this analysis–none of them statistically significant–were not discussed in the Executive Summary.

Subgroup analyses were conducted in 12 of the 13 studies (the exception being HOME-3). Evidence of spuriousness was found in all those studies, albeit to varying degrees. Table 3 summarises this evidence. Each column in Table 3 is a different study. Row 2 shows the number of subgroups included in the study. Rows 3 to 7 show the proportion of subgroup analyses meeting the criteria of spuriousness defined by Sun et al. [44]. Row 8 shows the average and standardised score of each study. Row 9 shows a qualitative assessment of the risk of spuriousness using the following rule: a score of 0.4 or less indicates a low risk; a score between 0.41 and 0.6 indicates a moderate risk and a score above 0.6 indicates a high risk. Table 3 shows that the subgroup analyses performed by the investigators had a low risk of spuriousness in two studies (DFE-2; HOM-2); a moderate risk in five studies (DFE-1; DFE-3; DWP-1; DWP-2; HOM-1) and a high risk in five studies (DFE-4; DWP-3; DWP-4; DWP-5; DWP-6). All subgroup analyses were based on data (i) collected or known at baseline and (ii) collected for that very study (as opposed to between-studies comparisons). Interactions were used in seven studies (DFE-1; DFE-2; DFE-3; DWP-1; DWP-2; HOME-1; HOM-2). Conversely, a theoretical justification for these analyses and an expected effect direction were very rarely mentioned. Some studies clearly indicated that these subgroup analyses were exploratory or ‘opportunistic’ (because the data was available) rather than confirmatory (Table 2, Quote 7).

**Table 3:** Spuriousness of subgroup analyses, based on Sun et al. (2010).

	DFE-1	DFE-2	DFE-3	DFE-4	DWP-1	DWP-2	DWP-3	DWP-4	DWP-5	DWP-6	HOM-1	HOM-2	HOM-3
Number of subgroups	4	5	5	7	4	10	9	4	4	10	4	6	0
A. Number of subgroup variables not measured at baseline*	0/4	1/5	0/5	0/7	0/4	0/10	0/9	0/4	0/4	0/10	0/4	0/6	0
B. Number of analyses suggested by comparisons of between studies (*vs*. within)	0/4	0/5	0/5	0/7	0/4	0/10	0/9	0/4	0/4	0/10	0/4	0/6	0
C. Number of subgroup analyses not based on interaction	0/4	0/5	0/5	7/7	0/4	0/10	9/9	4/4	4/4	10/10	0/4	0/6	0
D. No theoretical justification	4/4	2/5	5/5	7/7	4/4	10/10	9/9	4/4	4/4	10/10	4/4	2/6	0
E. Number of analyses for which the direction of the SG effect was not specified *a priori*	4/4	3/5	5/5	7/7	4/4	10/10	9/9	4/4	4/4	10/10	4/4	3/6	0
Average proportion (standardized)**	0.4	0.2	0.4	0.6	0.4	0.4	0.6	0.6	0.6	0.6	0.4	0.2	0
Overall risk of spuriousness	Medium	Low	Medium	High	Medium	Medium	High	High	High	High	Medium	Low	Nil

* This is the proportion of subgroup analyses based on data collected or known at baseline. For example, all subgroup analyses in study DFE-4 were based on data collected or known at baseline.

** This is the sum of all proportions for criteria A to E, divided by the number of criteria. For example, the score for study DFE-1 is: (0+0+0+1+1)/5 = 0.6.

Evidence of upgraded or downgraded outcomes was found in three of the 13 studies (DWP-3; DWP-6; HOME-2). In one study (HOME-2), the 11^th^ outcome of the intervention in the ‘results’ section became the ‘headline finding’ in the executive summary. This result happened to be the only one in the report showing a statistically significant effect of the intervention across all sites (see HOME-2, Figure 2.5, p.27). In another study (DWP-3), which found that benefit sanctions had had an insignificant effect on benefit off-flows (DWP-3, Table 4.18, p.29) and on employment entry (DWP-3, Table 4.19, p.29), the executive summary suggests that the aim of the study was actually to assess the *feasibility* of an impact evaluation (Table 2, Quote 8).

Evidence of conclusion bias was found in seven of the 13 studies (DFE-1; DFE-2; DFE-4; DWP-1; DWP-3; DWP-6; HOME-2). Among those seven studies, five were found to be highly spun (DFE-4; DWP-1; DWP-3; DWP-6; HOME-2). For example, the executive summary of the DWP-1 evaluation states, despite a primary outcome borderline non-significant at the 10% level, that the intervention was a success (Table 2, Quote 9). Two studies were found to be moderately spun (DFE-1; DFE-2). For example, the DFE-1 Executive Summary does acknowledge the non-significant result for the primary outcome of the study. However, this statement is immediately followed by another on the positive and significant effect of the intervention on one specific subgroup. The formulation might convey the idea that, overall, the intervention had a positive effect (DFE-1: p.4). In contrast, the executive summary of the DWP-2 study provides an example of language that was not considered ‘spun’ (Table 2, Quote 10).

### Effect of policy commitments

Looking first at the TS issued by commissioning departments, one can see a clear illustration of the cognitive dissonance that may be experienced by evaluators. On the one hand, tendering evaluators are required to provide evidence of their qualifications for the job (Quote 11). On the other hand, the document reminds the candidates that the policy and analysis teams within the commissioning departments will remain the ultimate decision-makers on key research decisions, including reporting (Quote 12). The dissonance problem is most obvious in Quote 13, in which the Department suggests that the government’s commitment to the intervention will have an effect on how evaluation outcomes will be reported. However, it is unclear from the above whether the association is positive (a stronger commitment leads to more spin) or negative (a stronger commitment leads to less spin). The notions of “high-quality reporting” and “policy relevant” are highly subjective. Quote 14, related to an intervention to which the government was strongly committed, suggests that the level of spin is limited.

Table 4 presents the overall risk of spin in the sample, broken down per type of spin and per study. As the risk of ORB through the omission of outcome indicators could not be assessed for all studies, the results were not included. The last three rows of Table 4 presents (i) a total risk of spin, which is the cumulated score of a given study based on the six indicators that could be assessed (from 0: minimum risk of spin to 6: maximum risk of spin); (ii) the ordinal variable used to measure the extent of the government’s commitment to the reform (from 1: pilot not announced to 5: pilot announced by the Prime Minister); and (iii) Pearson’s r coefficient for the two afore-mentioned variables. With r = -0.31, one can see that, to the extent that there is a linear correlation in the sample between the seniority of the reform’s champion and the risk spin, this correlation is negative and modest.

**Table 4:** Overall risk of spin per study and per type of spin.

	DFE-1	DFE-2	DFE-3	DFE-4	DWP-1	DWP-2	DWP-3	DWP-4	DWP-5	DWP-6	HOM-1	HOM-2	HOM-3	TOTAL
A. Missing outcome indicators	-	-	-	-	-	-	-	-	-	-	-	-	-	-
B. Incomplete reporting	0	0	1	0	0	0	0	1	1	0	1	0	1	5
C. Interpretative bias	0	0	0	1	1	0	0	1	0	0	0	1	1	5
D. Within-group comparisons	0	0	0	1	0	0	0	0	0	0	0	0	1	2
E. Subgroup analyses	1	1	1	1	1	1	1	1	1	1	1	1	0	12
F. Upgraded/ downgraded outcomes	0	0	0	0	0	0	1	0	0	1	0	1	0	3
G. Conclusion bias	1	1	0	1	1	0	1	0	0	1	0	1	0	7
Sum*	2	2	2	4	3	1	3	3	2	3	2	4	3	-
Endorser**	4	1	3	2	4	2	3	1	5	3	3	1	1	-
**Pearson’s r (Sum, Endorser)**	r = -0.31													

0 = This type of spin was not found in the study

1 = This type of spin was found

* Sum of criteria B to G (missing outcome indicators could not be recorded for all studies)

** 1 = The pilot was not announced; 2 = the pilot was announced by a junior minister; 3 = the pilot was announced by a senior minister; 4 = the pilot was announced by the Chancellor of the Exchequer; 5 = the pilot was announced by the Prime minister.

## 6. Discussion

This content analysis of 13 studies commissioned by the British government between 2007 and 2013 highlights two important findings. The first is that there is widespread evidence of ORB in the sample. All studies reviewed in this paper resorted to at least one of the seven presentational strategies associated by clinicians with a risk of spin. All departments are concerned. On average, nearly three different types of ORB were found in each study. The study presenting the lowest risk of ORB is DWP-2 and the studies presenting the highest risk of ORB are DFE-4 and HOME-2 (see Table 4). Moreover, all types of ORB were found in the sample. On average, a given type of ORB was found in between five and six studies. The least prevalent type of spin was the presentation of spurious within-group effects. The most prevalent type of spin was the presentation of spurious subgroup effects. This finding is in line with the rest of the literature, both in educational research [36] and in biomedical research [1,19]. Whether these spin strategies aim to deceive readers and to support pre-determined policies is a separate issue. In fact, this is not necessarily true, given that conclusion bias was not systematically associated with other types of spin. In other words, evidence of spin in long and fairly obscure ‘findings’ sections did not always result in spin in shorter and more policy-relevant ‘conclusion’ sections (or in executive summaries). This finding is consistent with the LSE GV314 survey, which points to a significant degree of docility at only the early stage in the researchers’ relationship with policy-makers, and at no other [64].

The second finding is that there is a small, negative association between the seniority of the reform’s champion and the risk of spin in the evaluation of that reform. In other words, pilots sponsored by senior ministers (e.g. Prime Minister, Chancellor) tend to be less spun than pilots sponsored by junior ministers or pilots that were not announced. The direction of this correlation is somewhat surprising and contrasts with the evidence on the effect of industry sponsorship on the reporting of clinical trial outcomes [72–74]. This literature suggests that higher (financial) stakes increase the risk of spin. The strength of this correlation is more difficult to interpret, given the lack of proper benchmark. However, it is safe to say that it is on the small side: pilots sponsored by senior ministers were *somewhat* less spun than pilots sponsored by junior ministers. This surprising association could have two explanations. The first explanation is based on capabilities: it could be that, on average, relatively salient pilots were better resourced and subject to more thorough reviews. The second explanation is based on a reverse reading of the ‘blame avoidance’ theory. Given that salient reforms are likely to attract scrutiny, it is safer for governments to present convincing results. Furthermore, one should bear in mind that spin can also occur at later stages of the policy cycle, notably in press releases and communications to parliament and stakeholders [75].

## 7. Implications

This concluding section looks at the theoretical, methodological and professional implications of the findings above.

### Theoretical implications

This study was based on the premise that spin in policy evaluation is (i) politically motivated, and (ii) commensurate with the salience of the reform. Whilst these theories cannot be completely rejected given the design of this study, two other directions should be investigated in future research. The first idea is that spin is not introduced by sponsors or research commissioners but by the researchers themselves. Both conscious and unconscious mechanisms are plausible. As already mentioned, the weakness of reporting norms means that researchers have no clear benchmark when they report their findings. However, the growing literature on data-mining (also known as p-hacking) would support the hypothesis of a conscious decision [13,76,77]. The second idea is that spin might actually flourish in the depths of low-level politics and in the evaluations of little-known programmes. If confirmed, this idea would give weight to the suggestions that (i) research should be more systematically audited; and (ii) governments should commission fewer but larger studies.

### Methodological implications

The qualitative approach taken in this study, combined with the small sample size, results in low external validity. Nevertheless, it sheds light on the feasibility of a large-N study on the prevalence of ORB in government-sponsored policy evaluation. This study highlights a few lessons for researchers attempting a scale up. First, the absence of formal research protocols in social research makes it impossible to compare what was reported with what would have been reported, had the outcome of the evaluation been favourable. Although technical specifications are useful documents, they are hard to obtain and not as specific as research protocols. As a result, the crucial question of missing outcomes cannot be addressed. Second, this research was hampered by the lack of consistency in the presentation of reports as well as the insufficient transparency in research decisions. Readability also proved to be an issue at times. To overcome these obstacles, investigators are advised to (i) work as a team; (ii) familiarise themselves with this type of literature before starting to collect data; and (iii) obtain missing information from authors and/or sponsors.

### Professional implications

Incomplete or misleading reports can impose significant costs on society: they can slow down the peer review process, delay the dissemination of important findings, increase the number of litigations between researchers and their sponsors, mislead evidence reviews and future research, mislead policy and hamper the teaching of evidence-based practice. Ultimately, they can tarnish the reputation of the research community as a whole. Two possible solutions are available to research sponsors and researchers concerned with transparency. The first is the publication and registration of research protocols. Repositories of study protocols have long been restricted to clinical trials, but evaluations of social interventions can now be registered as well, using for example 3ie’s *Impact Evaluation Repository* [78]. A second possible solution is the endorsement and use of reporting guidelines. Reporting guidelines are checklists, flow diagrams or explicit texts meant to guide authors in reporting their research [79]. Widely acknowledged guidelines include the Consolidated Standards for Reporting Trials (CONSORT) [58], the Preferred Reporting Items for Systematic reviews and Meta-Analyses (PRISMA) [59] and the Strengthening the reporting of observational studies in epidemiology (STROBE) [60].
